# Bacterial biofilms in the human body: prevalence and impacts on health and disease

**DOI:** 10.3389/fcimb.2023.1237164

**Published:** 2023-08-30

**Authors:** Elena K. Perry, Man-Wah Tan

**Affiliations:** Department of Infectious Diseases, Genentech, South San Francisco, CA, United States

**Keywords:** biofilm, aggregate, chronic infection, microbiome, adhesin, extracellular matrix, antibiotic tolerance, carcinogenesis

## Abstract

Bacterial biofilms can be found in most environments on our planet, and the human body is no exception. Consisting of microbial cells encased in a matrix of extracellular polymers, biofilms enable bacteria to sequester themselves in favorable niches, while also increasing their ability to resist numerous stresses and survive under hostile circumstances. In recent decades, biofilms have increasingly been recognized as a major contributor to the pathogenesis of chronic infections. However, biofilms also occur in or on certain tissues in healthy individuals, and their constituent species are not restricted to canonical pathogens. In this review, we discuss the evidence for where, when, and what types of biofilms occur in the human body, as well as the diverse ways in which they can impact host health under homeostatic and dysbiotic states.

## 1 Introduction

**Figure 1:**
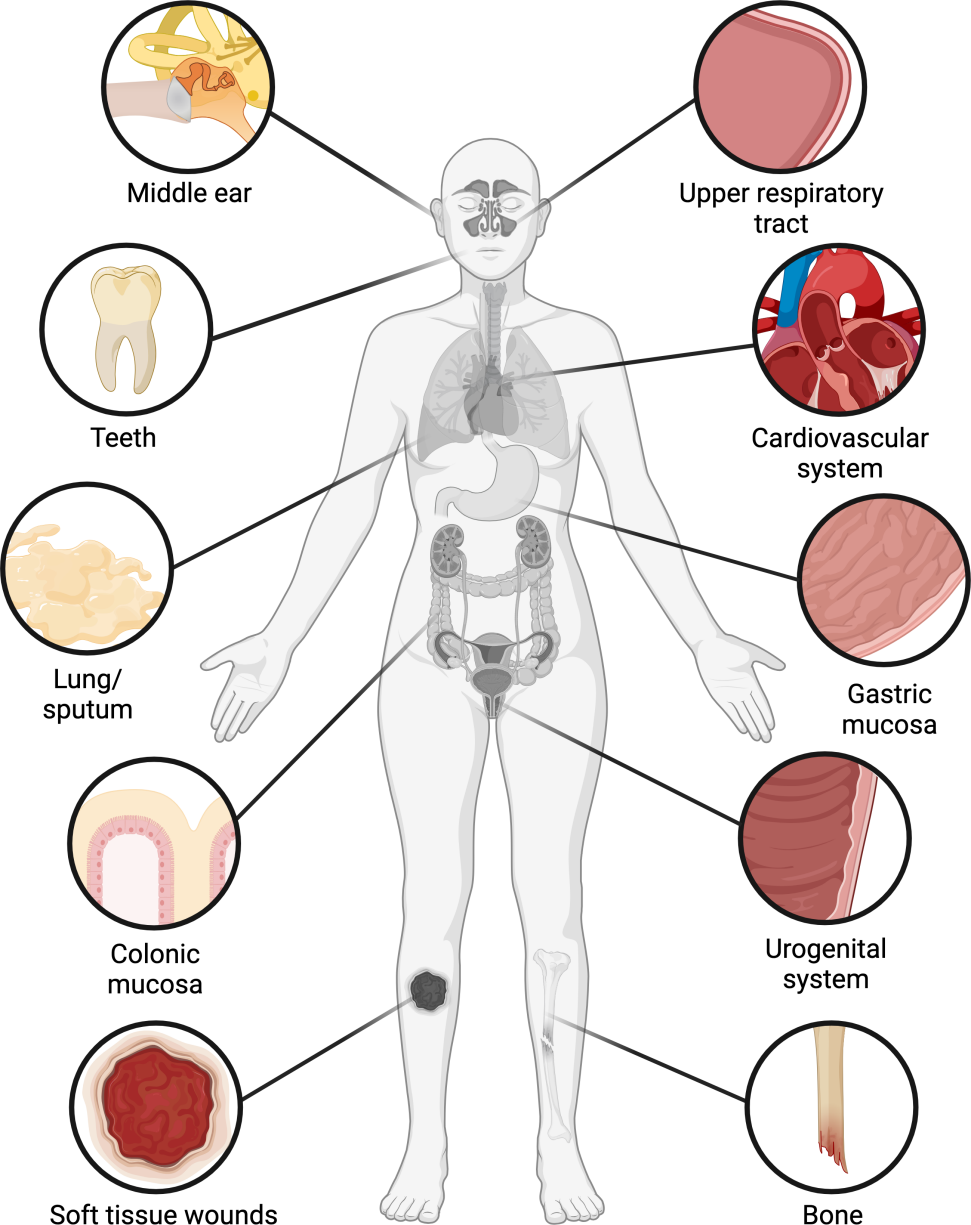
Occurrence of biofilms in the human body. Biofilms have been found in numerous organs and tissues, including the middle ear and upper respiratory tract, oral cavity, cardiovascular system, lung, stomach, colon, urogenital system, bone, and soft tissue wounds. In the oral cavity, colon, and female reproductive tract, biofilms occur with varying frequencies in clinically healthy patients as well as those with infections or underlying conditions, while in other tissues biofilms are generally associated with infections. Figure created with BioRender.com.

**Figure 2:**
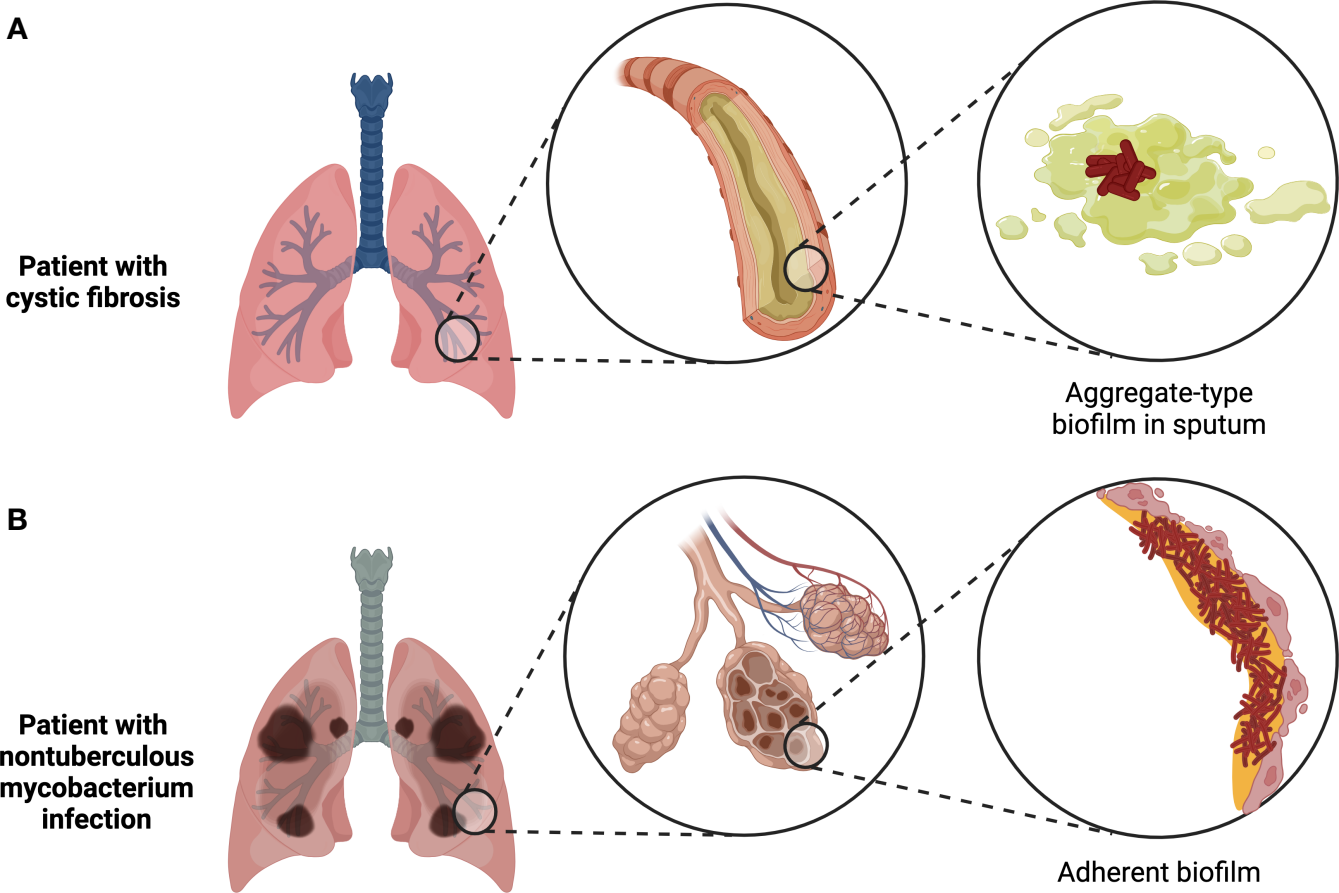
Two major types of biofilms found in the human body. Tissue-associated biofilms generally either take the form of bacterial aggregates suspended in mucus or other host secretions, as in the case of biofilms found in cystic fibrosis patient sputum **(A)**, or adherent biofilms attached to the surface of the tissue, as in the case of biofilms formed by nontuberculous mycobacteria during chronic infections **(B)**. Figure created with BioRender.com.

Bacteria have historically been studied as single-celled planktonic organisms, but outside of the laboratory, most bacteria live in organized multicellular communities embedded in a matrix of extracellular polymers, called biofilms (Flemming and Wuertz, 2019). Biofilms were arguably discovered as early as the 1680s, when Antonie van Leeuwenhoek described the presence of microorganisms and fibrous structures in the “scurf of the teeth” (i.e. dental plaque) (Leeuwenhoek, 1684). Yet it was not until the 1970s that the presence of bacterial biofilms began to be recognized in other sites within the human body (Høiby, 2017), most often through the use of techniques such as electron microscopy and fluorescent *in situ* hybridization (FISH) coupled with confocal laser scanning microscopy. Over the past few decades, biofilm-containing samples have been acquired from numerous tissues that are classically thought to be sterile, where biofilms are generally associated with infections (**Figure 1**, **Table 1**). Biofilms have also been found on tissues that have long been known to harbor commensal microbiota, where the biofilms may or may not be pathogenic (**Figure 1**, **Table 1**). In some types of samples, biofilms primarily occur as aggregates suspended in mucus or other host secretions (**Figure 2A**, **Table 1**). In others, the biofilms are attached to the tissue itself—typically at a mucosal interface, although there are exceptions (**Figure 2B**, **Table 1**). In spite of their morphological differences, suspended aggregates and surface-attached bacterial communities share many functional properties, suggesting that both types of biofilms represent fundamentally similar modes of bacterial existence (Alhede et al., 2011).

**Table 1:** Key evidence for the presence of biofilms in the human body.

Location	Methods	Observations	Common species	Associated conditions	Key references
Lower respiratory tract	Light microscopy; FISH and confocal microscopy	Bacterial aggregates suspended in sputum; adherent bacteria in necrotic lung cavities (in mycobacterial infections)	*P. aeruginosa*, *S. aureus*, *Streptococcus* spp., *H. influenzae*, *M. catarrhalis*, *Mycobacteria* spp.	Chronic infection of cystic fibrosis patients; bronchiectasis; protracted bacterial bronchitis; acute pneumonia	Bjarnsholt et al., 2009; DePas et al., 2016; Fennelly et al., 2016; Basaraba and Ojha, 2017
Upper respiratory tract	Scanning electron microscopy; FISH and confocal microscopy	Adherent biofilms on the sinus mucosa	*H. influenzae, S. aureus*, *S. pneumoniae*	Chronic rhinosinusitis	Sanderson et al., 2006; Bezerra et al., 2011
Middle ear	Scanning electron microscopy; FISH and confocal microscopy	Clusters of bacteria in aspirated secretions; adherent bacterial colonies on mucosal biopsies	*H. influenzae*, *M. catarrhalis*, *S. pneumoniae*, *K. pneumoniae*, *S. aureus*	Chronic otitis media	Hall-Stoodley et al., 2006; Homøe et al., 2009; Lee et al., 2009
Soft tissue wounds	Light microscopy; scanning and transmission electron microscopy; FISH and confocal microscopy	Bacterial aggregates embedded in the wound bed or on the wound surface	*P. aeruginosa*, *S. aureus*, *Enterococcus* spp., *Enterobacter* spp., *Proteus mirabilis*	Delayed healing; predispositions include diabetes and severe burns	James et al., 2008; Kennedy et al., 2010; Fazli et al., 2011
Urinary tract	Light microscopy; scanning and transmission electron microscopy; immuno- fluorescence	Intracellular and extracellular clusters of bacteria in urine; matrix-encased clusters of bacteria within kidney stones and adhering to the stone surface	*E. coli*, *P. aeruginosa*, *P. mirabilis*, *Staphylococcus* spp., *Enterococcus* spp.	Urinary tract infection; kidney stones	Nickel et al., 1985; Rosen et al., 2007; Romanova et al., 2015
Male reproductive tract	Scanning and transmission electron microscopy	Microcolonies adhering to the prostate ductal wall	*E. coli*, *P. aeruginosa*, *Staphylococcus* spp.	Chronic prostatitis	Nickel and Costerton, 1993
Female reproductive tract	Transmission electron microscopy; FISH and fluorescence or confocal microscopy	Bacterial aggregates in vaginal secretions; biofilms adhering to the vaginal epithelium	*Lactobacillus* spp., *G. vaginalis*, *F. vaginae*	Bacterial vaginosis	Swidsinski et al., 2005a; Hardy et al., 2015
Cardiovascular system	Scanning and transmission electron microscopy; FISH and confocal microscopy	Large clusters of bacteria encased within vegetations on heart valves; biofilm-like microcolonies within atherosclerotic arterial tissue and between the vascular smooth muscle and luminal plaque	*S. aureus*, *Streptococcus* spp., *Enterococcus* spp.	Infective endocarditis, atherosclerosis	Marrie et al., 1987; Mallmann et al., 2010; Lanter et al., 2014; Snow et al., 2016
Bone	Scanning electron microscopy; FISH and confocal microscopy	Thick, dense biofilms covering large areas of the bone surfaces	*S. aureus*, *P. aeruginosa*, *Streptococcus* spp.	Osteomyelitis	Gristina et al., 1985; Johani et al., 2019
Oral cavity	FISH and fluorescence or confocal microscopy	Highly structured multispecies biofilms collected from teeth	*Streptococcus* spp., *Actinomyces* spp., *Veillonella* spp., *Fusobacterium* spp., *Porphyromonas* spp., *Aggregatibacter* spp.	Dental caries, periodontal disease	Zijnge et al., 2010; Welch et al., 2016
Stomach	Scanning electron microscopy	Extensive biofilms covering the gastric mucosa	*H. pylori*	Peptic ulcers and gastric cancer	Carron et al., 2006; Coticchia et al., 2006
Colon	Light microscopy; FISH and confocal microscopy	Dense, adherent multispecies biofilms on the intestinal epithelium	*E. coli*, *Bacteroides* spp., *R. gnavus*, *F. nucleatum*	Colorectal cancer, inflammatory bowel disease, irritable bowel syndrome, post organ transplantation	Swidsinski et al., 2005b; Dejea et al., 2014; Baumgartner et al., 2021

In this review, we first describe where biofilms have been found in the human body and under what circumstances, as well as which taxa are present; we focus on tissue-associated biofilms rather than biofilms associated with implanted medical devices, which have been recently reviewed elsewhere (Khatoon et al., 2018; Yadav et al., 2020). We then summarize what is known about surface-associated and secreted polymers involved in biofilm formation by human pathogens and commensal bacteria, and how environmental factors influence biofilm development *in vivo*. We present an overview of the ways in which biofilms have been proposed to impact human health, including supporting evidence. Finally, we discuss key limitations of current knowledge about tissue-associated biofilms and highlight the importance of developing novel experimental models to address major open questions in the field.

## 2 Occurrence of biofilms in the human body

### 2.1 Infection-associated biofilms

One of the longest-recognized sites of infection-associated biofilms is the lower respiratory tract, where the discovery of bacterial aggregates suspended in sputum was first reported in chronically-infected cystic fibrosis (CF) patients 50 years ago (Høiby and Axelsen, 1973; Høiby, 2017). Intriguingly, recent evidence suggests that aggregate-type biofilms in sputum may be common not only in chronic lung infections, as is now well-accepted, but also in acute respiratory infections, where planktonic bacteria have long been assumed to be the dominant form (Kolpen et al., 2022). However, virtually all investigations of biofilms in the lung to date have focused on chronic infections. The most common biofilm-forming pathogen found in CF patients is *P. aeruginosa* (Bjarnsholt et al., 2009; Williams and Davies, 2012), but aggregates of *Staphylococcus* or *Streptococcus* species have also been detected in optically-cleared CF patient sputum samples using genus-specific FISH probes (DePas et al., 2016). In the latter study, *Pseudomonas* occurred as a bimodal mix of small and large aggregates (<50 µm^3^ or >1000 µm^3^, respectively), while *Staphylococcus* occurred in small to medium-sized aggregates (50-1000 µm^3^), and *Streptococcus* primarily existed in large aggregates (>1000 µm^3^), some of which encased host cells. Besides the taxa that have been definitively detected by FISH in CF sputum biofilms, strains of *Burkholderia cepacia* complex species isolated from CF patients are frequently proficient at forming biofilms *in vitro* and may also be capable of forming biofilms *in vivo* (Cunha et al., 2004); the lack of attempts at direct detection of *Burkholderia* in CF sputum biofilms by FISH to date may be due to the relatively low prevalence of this clade in CF infections (Salsgiver et al., 2016). The observations published thus far suggest that individual bacterial aggregates in CF sputum tend to be monospecies (Bjarnsholt et al., 2009; DePas et al., 2016), even though patients can be co-infected by multiple species simultaneously and mixed-species biofilms can be formed by common CF pathogens *in vitro* (Pompilio et al., 2015; Magalhães et al., 2022). In addition, while most biofilms in CF patients are thought to occur in sputum, biofilms of *Mycobacterium abscessus* have been detected in the interstitial spaces of the alveolar wall in chronically-infected CF patients (Qvist et al., 2015).

Beyond CF patients, lower airway biofilms have been detected in other long-term respiratory infections, such as bronchiectasis and protracted bacterial bronchitis, where the represented species include *Haemophilus influenzae, Moraxella catarrhalis*, *P. aeruginosa, Streptococcus pneumoniae*, and *Staphylococcus* species (Marsh et al., 2022). As with the biofilms found in CF patients, other lung infection-associated biofilms typically occur as suspended aggregates in sputum (Kolpen et al., 2022). However, adherent biofilms may play a role in the pathogenesis of chronic pulmonary infections by nontuberculous mycobacteria, given that *M. abscessus* biofilms were observed lining a resected lung cavity from an infected patient with chronic obstructive pulmonary disease (Fennelly et al., 2016). *Mycobacterium tuberculosis* has similarly been proposed to form extracellular biofilms in necrotic lesions and lung cavities (Orme, 2014; Basaraba and Ojha, 2017). Indeed, dense masses and sheets of bacteria that morphologically resemble biofilms have been directly observed in resected lung sections from tuberculosis patients (Nyka, 1963; Nyka and O’Neill, 1970). More recently, clusters of mycobacterial cells in tuberculosis-infected human lungs were shown to be encased by an exopolysaccharide-containing matrix, further supporting their biofilm-like character (Chakraborty et al., 2021). Nevertheless, the overall contribution of biofilm formation to mycobacterial pathogenesis remains poorly understood.

Infection-associated biofilms have also been detected in the middle ear and upper respiratory tract, which are connected by the eustachian tube. In the middle ear, biofilms ranging from microcolonies to large clusters of bacteria have been detected at a high frequency in patients with chronic otitis media, both in aspirate samples and mucosal biopsies, but only very rarely in patients with no known history of ear infection (Hall-Stoodley et al., 2006; Homøe et al., 2009; Lee et al., 2009). Pathogens detected in chronic otitis media-associated biofilms include *H. influenzae*, *M. catarrhalis*, *S. pneumoniae*, *Klebsiella pneumoniae*, and *Staphylococcus aureus*, all of which are also common colonizers of the upper respiratory tract. Similar to the biofilms observed in lung sputum samples, discrete biofilms observed in chronic otitis media often appear to be monospecies, even in patients co-infected with multiple pathogens (Hall-Stoodley et al., 2006). Elsewhere in the upper respiratory tract, biofilms have been detected in the sinus mucosa, adenoids, and tonsils (Morris, 2007)—typically in patients with chronic rhinosinusitis or recurrent upper respiratory tract infections, although some studies also reported the presence of putative biofilms in a smaller proportion of patients with non-infectious nasal obstruction (Sanderson et al., 2006; Bezerra et al., 2011).

Outside of the respiratory tract, several studies have reported the presence of bacterial biofilms in chronic or slow-healing soft tissue wounds (Bjarnsholt et al., 2008; James et al., 2008; Kennedy et al., 2010; Fazli et al., 2011; Han et al., 2011; Percival et al., 2015). Wound-colonizing biofilms range in appearance from microcolonies to aggregates to thick, continuous biofilms (Metcalf and Bowler, 2013; Percival et al., 2015). The latter may even be macroscopically visible in some cases (Metcalf and Bowler, 2013), although this remains a point of controversy in the field (White and Cutting, 2012). Biofilms in chronic wounds may be either monospecies or multispecies, with two of the most commonly detected species being *P. aeruginosa* and *S. aureus* (Metcalf and Bowler, 2013; Percival et al., 2015). Biofilms have also been found in bone infections (osteomyelitis), which usually follow trauma or occur in tissues that are otherwise compromised, such as through vascular damage (Gristina et al., 1985; Sedghizadeh et al., 2009). Scanning electron microscopy of samples from patients with osteomyelitis of the femur, tibia, or fibula revealed thick, extensive multispecies biofilms coating the surface of the bone, while transmission electron microscopy revealed microcolonies attached to host cells derived from the surrounding tissue (Gristina et al., 1985). The biofilms appeared to be multispecies, due to the presence of different morphotypes such as cocci and rods, and culture results revealed a mixture of species in most patients, including *S. aureus*, *P. aeruginosa*, *Serratia marcescens*, and *Bacteroides* species (Gristina et al., 1985). Scanning electron microscopy and confocal microscopy similarly revealed dense aggregates of bacteria on the bone surface in patients with diabetic foot osteomyelitis (Johani et al., 2019). Most of the observed biofilms appeared to be multispecies based on the presence of both cocci and rods, and 16S rRNA gene amplicon sequencing revealed that the most abundant genera included *Corynebacterium*, *Finegoldia*, *Staphylococcus*, *Streptococcus*, *Porphyromonas*, and *Anaerococcus* (Johani et al., 2019). Biofilms were also present in patients with osteomyelitis or osteonecrosis of the jawbone, covering large areas of both the internal and external surfaces of the bone (Sedghizadeh et al., 2009). Based on examination of the bacterial morphology by scanning electron microscopy, the biofilms in the jawbone osteomyelitis patients appeared to be predominantly comprised of *Actinomyces*, a genus commonly found in the oral cavity, while the biofilms in osteonecrosis were multispecies and contained morphotypes consistent with other typical oral bacteria such as *Fusobacterium* and *Streptococcus*, in addition to *Actinomyces* (Sedghizadeh et al., 2009).

In the urogenital system, extracellular-matrix-encased microcolonies of adherent bacteria have been detected in biopsies from male patients with chronic prostatitis (Nickel and Costerton, 1992; Nickel and Costerton, 1993). In addition, *Escherichia coli* has been shown in a mouse model to form biofilm-like “pods” inside bladder epithelial cells during urinary tract infections (Anderson et al., 2003). Although invasive biopsies are not typically performed for urinary tract infections, analysis of urine from actively infected patients revealed biofilm-like bacterial aggregates and putative intracellular bacterial communities within exfoliated host cells, suggesting that similar mechanisms of pathogenesis also occur in humans (Rosen et al., 2007). Higher up in the urinary tract, biofilms have been observed on the surface and in the interior of kidney stones (Nickel et al., 1985; McLean et al., 1989; Romanova et al., 2015). Typical organisms associated with kidney stone biofilms include *P. aeruginosa*, *E. coli*, *K. pneumoniae*, *Proteus mirabilis*, *Staphylococcus* species, and *Enterococcus* species (Nickel et al., 1985; Romanova et al., 2015). Some of these species possess urease activity (Romanova et al., 2015), which precipitates the formation of specific minerals found in kidney stones (McLean et al., 1989). Moreover, biofilm extracellular matrix components have been proposed to play a role in cementing nascent mineral crystals during kidney stone formation (McLean et al., 1989).

Finally, biofilms have been detected in the circulatory system in the context of infections of the heart (endocarditis) and in atherosclerotic arteries. Infective endocarditis occurs when bacteria—most often *S. aureus*, *Streptococcus* species, or *Enterococcus* species—enter the bloodstream and subsequently attach to the heart valves or inner lining of the heart chambers, typically in patients with congenital valve abnormalities or damaged heart tissue (Cabell et al., 2003; Lerche et al., 2021). During the course of disease progression, irregular masses of tissue form on the heart lining, which are called “vegetations” (Cabell et al., 2003; Lerche et al., 2021). Examination of vegetations by transmission electron microscopy revealed masses of bacteria encased by an extracellular matrix (Marrie et al., 1987). Large biofilm-like clusters of bacteria within infected heart valve tissue have also been identified using FISH and confocal microscopy on biopsies from endocarditis patients (Mallmann et al., 2010). Similarly, in atherosclerotic arteries, FISH performed with a universal bacterial probe revealed the presence of biofilm-like microcolonies of bacteria both within the arterial tissue and between the vascular smooth muscle and the luminal plaque (Lanter et al., 2014; Snow et al., 2016).

### 2.2 Tissues with non-infection-associated biofilms

In organ systems that are colonized by a commensal microbiota in healthy individuals, such as the female reproductive tract and gastrointestinal tract, the presence of a biofilm is not always correlated with infection or injury. For example, loosely-attached conglomerates of commensal *Lactobacillus* species, which potentially represent aggregate-type biofilms, can sometimes be found in vaginal biopsies from healthy patients (Swidsinski et al., 2005a). On the other hand, dense biofilms that firmly adhere to the vaginal wall and mainly consist of *Gardnerella vaginalis* and *Fannyhessea vaginae* are characteristic of bacterial vaginosis—yet such biofilms are also found in a minority of asymptomatic individuals (Swidsinski et al., 2005a; Hardy et al., 2015). In the gastrointestinal tract, oral biofilms are by far the most extensively studied due to the accessibility of samples and the fact that dental plaque—a form of multispecies biofilm—is ubiquitous even in healthy individuals. The diversity of oral bacteria in humans spans at least several hundred species and nine phyla (Zijnge et al., 2010), and different community compositions have been associated with healthy versus disease states (e.g. periodontitis) (Socransky et al., 1998; Griffen et al., 2012). Nevertheless, the development of dental plaque appears to follow a reproducible general pattern of ecological succession: *Streptococcus*, *Actinomyces*, and *Veillonella* dominate early plaque, followed by an increase in the proportion of *Fusobacterium* and *Porphyromonas*, and finally the advent of late colonizers such as *Aggregatibacter*, which depend on the presence of other species to create a suitable niche (Kolenbrander and London, 1993; Palmer et al., 2003; Al-Ahmad et al., 2007; Periasamy and Kolenbrander, 2010). In addition, the spatial structure of dental plaque is highly organized (Zijnge et al., 2010; Welch et al., 2016), likely reflecting environmental and biochemical gradients in combination with physical and metabolic interactions between the constituent organisms.

**Figure 3:**
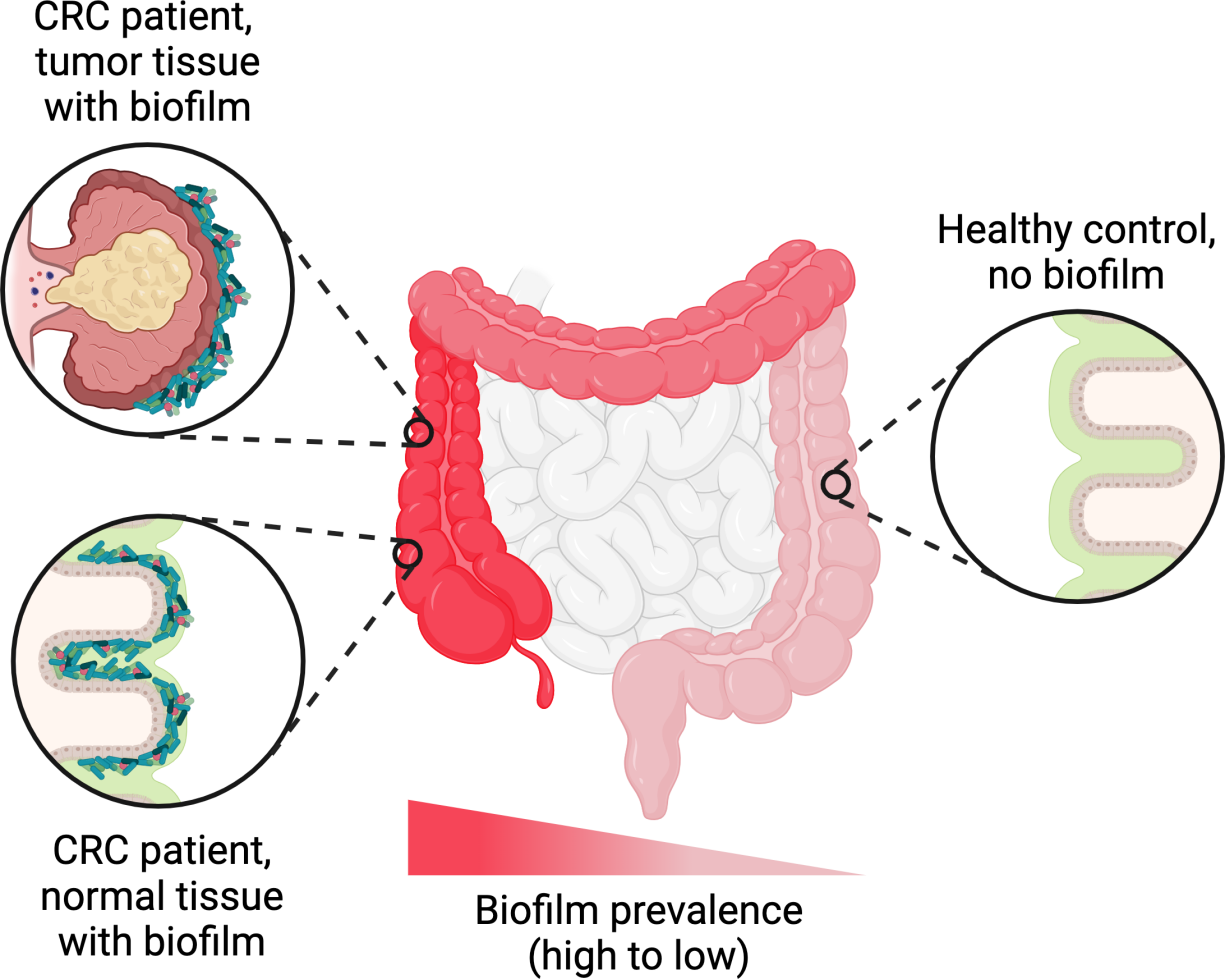
Trends in biofilm occurrence in the distal gastrointestinal tract. Adherent biofilms on the colonic epithelium are found in a higher proportion of patients with conditions such as colorectal cancer (CRC), inflammatory bowel disease, or irritable bowel syndrome compared to healthy controls. In biofilm-positive CRC patients, the biofilms are generally not restricted to the tumor itself and can also be found on normal colonic tissue. Regardless of the patient’s underlying condition, the frequency of biofilm detection follows a decreasing trend along the longitudinal axis of the colon, with higher frequencies in the ascending colon compared to the transverse and descending colon. Figure created with BioRender.com.

Further down in the gastrointestinal tract, extensive biofilms of *Helicobacter pylori* have been observed on the gastric mucosa of infected patients (Carron et al., 2006; Coticchia et al., 2006). In the ileum and colon, biofilms containing dense masses of bacteria in close contact with the epithelial surface are detectable in a minority of healthy people (6-35% depending on the study) (Swidsinski et al., 2005b; Dejea et al., 2014; Baumgartner et al., 2021). However, in dysbiotic states such as colorectal cancer, inflammatory bowel disease, or irritable bowel syndrome, biofilms are observed more frequently on the intestinal epithelium—sometimes in up to 50% of patients or even higher (Swidsinski et al., 2005b; Dejea et al., 2014; Baumgartner et al., 2021). Biofilms may also represent a reservoir for certain canonical intestinal pathogens; for example, *Salmonella* spp. form biofilms on gallstones in asymptomatic human carriers (Crawford et al., 2010). Remarkably, while most of the evidence for intestinal biofilms comes from microscopic examination of biopsies, some biofilms may be macroscopically visible during endoscopies (Baumgartner et al., 2021). Such biofilms have been reported in the upper jejunum of patients diagnosed with small intestinal bacterial overgrowth, as well as in the ileum and colon of patients with other disorders (Baumgartner et al., 2021). In accordance with the complexity of the overall gut microbiota, intestinal biofilms are almost invariably polymicrobial, although they tend to be enriched in certain taxa. These taxa vary depending on the study, likely reflecting differences in patient population and/or etiology, but generally include *E. coli*, *Bacteroides* species, certain Clostridia such as *Ruminococcus gnavus*, and/or oral pathogens such as *Fusobacterium nucleatum* (Swidsinski et al., 2005b; Dejea et al., 2014; Drewes et al., 2017; Baumgartner et al., 2021). Multiple studies have also reported that biofilms are much more common near the ileal-cecal junction and in the ascending colon compared to the transverse colon, descending colon, or rectum, regardless of the patient’s underlying condition (Dejea et al., 2014; Drewes et al., 2017; Baumgartner et al., 2021) (**Figure 3**). The mechanism driving this skewed longitudinal distribution remains unclear, but presumably is related to physical and/or chemical differences along the length of the colon.

## 3 Intrinsic and extrinsic factors affecting tissue-associated biofilm development

While biofilms by definition consist of sessile bacteria, they are not static structures. Rather, they undergo a dynamic and often cyclical process of development and change (Stoodley et al., 2002). Broadly speaking, regardless of the specific form a biofilm takes, the biofilm life cycle encompasses three stages: aggregation and attachment, growth and accumulation, and disaggregation and detachment (Sauer et al., 2022). Each of these steps is influenced both by characteristics intrinsic to the bacteria themselves, such as cell surface architecture, and by extrinsic signals, substrates, and physical forces in their environmental milieu.

### 3.1 Surface polymers involved in the initial steps of biofilm formation

The first step in biofilm formation—aggregation and attachment—is largely driven by structures on the bacterial cell surface. The diversity and number of adhesive factors precludes a comprehensive review of every known bacterial adhesin; rather, here we aim to offer a general overview of the types of cell surface structures that have been implicated in adherence to host tissues or the formation of aggregates within the host environment (**Table 2**). All types of biofilms, including suspended aggregates, involve adhesive interactions between bacterial cells, while tissue-attached biofilms also require adhesins that mediate bacteria-substrate interactions. Some adhesins are thought to predominantly contribute to only one of these types of interactions, but many have been shown to contribute to both. Indeed, experiments that demonstrate the role of an adhesin in attachment to a particular substrate do not necessarily rule out a secondary role in mediating bacterial intercellular adhesion, suggesting that adhesins with dual roles could be even more widespread than is currently evident.

**Table 2:** Major classes of bacterial adhesins.

Category	Named examples	Species	Binding substrates
Chaperone-usher pili	Type I pilus, Saf, *E. coli* common pilus, F1C fimbriae	Enteric bacteria	Epithelial and endothelial cells, self-aggregation
Type IV pili	–	*P. aeruginosa*, *E. coli*, *S. sanguinis*	Epithelial cells
Type V pili	–	*Porphyromonas gingivalis*, *Bacteroides* spp.	Host extracellular matrix proteins, epithelial cells
Amyloid-like adhesins	Curli, FadA	Enteric bacteria, *F. nucleatum*, *M. tuberculosis*	Fibronectin, epithelial and endothelial cells, self-aggregation
Sortase-assembled pili	SpaC	*Streptococcus* spp., *Lactobacillus* spp.	Saliva-coated surfaces, epithelial cells, mucins
MSCRAMMs	ClfA/B, FnBPs, Cna, RadA	*Staphylococcus* spp., *Streptococcus* spp., *R. gnavus*, *Enterococcus faecalis*	Host extracellular matrix proteins, glycoproteins
Proteins with G5-E repeat domains	SasG	*Staphylococcus* spp.	Epithelial cells, self-aggregation
Lectin-like proteins	SraP, Fap2	*Staphylococcus* spp., *F. nucleatum*	Epithelial cells
Serine-rich repeat proteins	Srr1, Srr2	*Streptococcus* spp.	Saliva-coated surfaces, fibrinogen
Lipoproteins	LraI, Slr	*Streptococcus* spp.	Saliva-coated surfaces, collagen, other bacteria
Outer membrane proteins	OmpF, HBP35, FomA	*P. aeruginosa*, *P. gingivalis*, *F. nucleatum*	Epithelial cells, saliva-coated surfaces, other bacteria
Flagellin	FliC	*P. aeruginosa*, *E. coli*	Mucins
Autotransporters	AIDA, Ag43, TibA, RadD	Enteric bacteria, *F. nucleatum*	Epithelial cells, self-aggregation, other bacteria
Extracellular polysaccharides	Poly-*N*-acetylglucosamine, Pel, Psl, glucans	*E. coli*, *S. aureus*, *P. aeruginosa*, *Streptococcus* spp.	Epithelial cells, mucins, saliva-coated surfaces, self-aggregation

#### 3.1.1 Pili and pili-like structures

Among the proteinaceous structures involved in bacterial adhesion and aggregation, pili are perhaps the best studied. Sometimes referred to as fimbriae, pili are non-flagellar cell-surface appendages with a modular structure composed of individual protein subunits. Several distinct types of pili have been described thus far, including chaperone–usher pili, type IV pili, type IV secretion pili, type V pili, curli fibers, and sortase-assembled pili (Telford et al., 2006; Hospenthal et al., 2017). Different classes of pili vary in their structure, biochemistry, and biogenesis, but virtually all have been found to play roles in bacterial adhesion except for type IV secretion pili, which instead facilitate conjugative transfer of genetic material between bacterial cells (Hospenthal et al., 2017).

Chaperone-usher pili are so named because of the manner of their biogenesis, which involves chaperone-mediated folding of pilin subunits in the periplasm followed by polymerization and secretion through an “usher” protein (Busch and Waksman, 2012). Pili belonging to this diverse class were the first to be recognized as being involved in bacterial cell adhesion (Duguid et al., 1955; Busch and Waksman, 2012) and are commonly found in Gram-negative pathogens, especially among enteric bacteria (Yen et al., 2002; Zav’yalov et al., 2010). Chaperone-usher pili can either be monoadhesive, meaning that only the subunit at tip of the pilus contains a binding domain that interacts with host tissues or other substrates, or polyadhesive, meaning that each individual subunit is capable of mediating adhesion (Zav’yalov et al., 2010). In both cases, the biochemical properties of the binding domain can confer tropism for specific host tissues. For example, the FimH domain of the monoadhesive type I pilus expressed by uropathogenic *E. coli* binds to mannosylated uroplakin UP1a and α1β3 integrins on umbrella cells in the bladder epithelium (Eto et al., 2007; Proft and Baker, 2008), while the polyadhesive Saf pilus of *Salmonella enterica* mediates binding to intestinal epithelial cells (though the host receptor remains unknown) (Zeng et al., 2017). Other chaperone-usher pili likely involved in biofilm formation *in vivo* include the *E. coli* common pilus, or ECP, and F1C fimbriae (Wurpel et al., 2013). ECP is ubiquitous across *E. coli* and has been implicated in adherence to epithelial cells and intestinal colonization by both pathogenic and commensal strains (Lasaro et al., 2009; Avelino et al., 2010). F1C fimbriae are expressed by a much smaller subset of *E. coli* strains but have been shown to bind specific host glycolipids (Khan et al., 2000), mediate adhesion to endothelial cells in the human kidney and bladder (Virkola et al., 1988), and contribute to persistent intestinal colonization in a mouse model (Lasaro et al., 2009). Notably, while most studies on chaperone-usher pili tropisms have focused on binding to host substrates, some pili in this class may also mediate bacteria-bacteria interactions during biofilm formation through pilus self-association. This has been demonstrated at least for Saf in *S. enterica*, in which mutations that disrupted pilus self-association also hindered biofilm formation (Zeng et al., 2017). ECP is also likely to be involved in mediating adhesion between bacterial cells, as scanning electron micrographs of *E. coli* microcolonies revealed complex meshes of intertwined ECP fibers (Garnett et al., 2012).

Type IV pili are the most broadly distributed type of pilus and the only class that has been found in both Gram-negative and Gram-positive bacteria (Pelicic, 2008). Characterized by their particularly thin (5-9 nm diameter) and long (up to several micrometers) morphology (Pelicic, 2008; Hospenthal et al., 2017), type IV pili are thought to be the dominant factor mediating adhesion of *P. aeruginosa* to human airway epithelial cells (Hahn, 1997), even though they are not always necessary for attachment to abiotic surfaces (Klausen et al., 2003). This adhesion may result from binding of the pilus tip to specific glycolipids (Lee et al., 1994). Beyond *P. aeruginosa*, type IV pili contribute to localized adherence of enteropathogenic *E. coli* to host cells (Donnenberg et al., 1992) and epithelial cell adhesion and platelet-mediated biofilm formation by *Streptococcus sanguinis* (Chen et al., 2019; Martini et al., 2021). Type IV pili expressed by the intestinal bacteria *Clostridium perfringens* and *Clostridioides difficile* likewise appear to be involved in biofilm formation at least *in vitro* (Varga et al., 2008; Maldarelli et al., 2016). As with chaperone-usher pili, type IV pili can mediate bacteria-bacteria interactions and auto-aggregation in addition to interactions with host substrates (Vuopio-Varkila and Schoolnik, 1991; Bordeleau et al., 2015).

Type V pili are unique to bacteria in the class Bacteroidia and have only been defined structurally and biochemically within the last decade (Xu et al., 2016), although their existence has been recognized since the 1980s (Yoshimura et al., 1984). Type V pili expressed by the oral pathogen *Porphyromonas gingivalis* mediate binding to gingival epithelial cells and human extracellular matrix proteins via a multitude of adhesion epitopes (Hajishengallis, 2007), in addition to promoting coaggregation with *Streptococcus gordonii* and potentially other oral bacteria (Park et al., 2005). A putative type V pilus also contributes to *in vitro* biofilm formation by the gut commensal *Bacteroides thetaiotaomicron* (Mihajlovic et al., 2019). It is therefore tempting to speculate that type V pili could be involved in the formation of the *Bacteroides*-enriched biofilms observed in some inflammatory bowel disease and colorectal cancer patients (Swidsinski et al., 2005b; Drewes et al., 2017). Variants of this pilus that either promoted or diminished biofilm formation *in vitro* did not affect colonization of germ-free mice by *B. thetaiotaomicron* (Mihajlovic et al., 2019). However, its role in adhering to human epithelial cells or mucus has not been directly tested for *Bacteroides* species, and the possibility remains that type V pili might contribute to co-aggregation with other biofilm-forming bacteria in the gut, or only be essential for adhesion to the intestinal mucosa under certain circumstances.

Other types of pili or fimbriae that can contribute to adhesion to host cells and/or human extracellular matrix include extracellular amyloid fibers called curli that are produced by many strains of *E. coli* and *Salmonella* species (Barnhart and Chapman, 2006), the fimbrial protein FadA in *F. nucleatum* (Han et al., 2005; Fardini et al., 2011), and sortase-assembled pili found in Gram-positive bacteria (Telford et al., 2006). Curli mediates bacterial auto-aggregation and binding to fibronectin, and works synergistically with other bacterially-produced extracellular matrix components to promote adherence to human intestinal epithelial cells (Collinson et al., 1993; Saldaña et al., 2009). A curli-like pilus is also produced by *M. tuberculosis* during human infection, and mutants lacking this pilus are deficient in laminin binding and *in vitro* biofilm formation (Alteri et al., 2007; Ramsugit et al., 2013). FadA is a unique amyloid-like fimbrial adhesin found only in *Fusobacterium* species, which is required for adhesion to human epithelial and endothelial cells (Han et al., 2005; Fardini et al., 2011; Meng et al., 2021). Sortase-assembled pili expressed by Gram-positive oral pathogens can mediate adhesion either to saliva-coated tooth surfaces or host cells, while the pili produced by *Streptococcus pneumoniae* and *Streptococcus agalactiae* promote adhesion to lung epithelial cells, and pili produced by *Streptococcus pyogenes* bind to collagen (Telford et al., 2006; Konto-Ghiorghi et al., 2009). In the case of at least *S. agalactiae*, the pilus is also essential for adherent biofilm formation on plastic surfaces, but in a manner that is independent of the von Willebrand adhesion domain that mediates epithelial cell adhesion, suggesting that different domains of the pilus confer different surface tropisms (Konto-Ghiorghi et al., 2009). Finally, the sortase-assembled pilus of the probiotic bacterium *Lactobacillus rhamnosus* GG contains a specific subunit, SpaC, that binds β-galactoside-containing carbohydrate moieties in human intestinal mucus (Kankainen et al., 2009; Nishiyama et al., 2016), in addition to mediating auto-aggregation, collagen binding, and adhesion to intestinal epithelial cells (though the latter could be a result of binding mucins attached to the epithelial cell surface) (Tripathi et al., 2013; Ardita et al., 2014).

#### 3.1.2 Non-pili-mediated adhesion

Besides pili and fimbriae, numerous other bacterial adhesive factors have been described. Gram-positive cocci such as *Staphylococcus*, *Streptococcus*, and *Enterococcus* spp. express a diverse range of cell-wall anchored proteins involved in intercellular adhesion or adhesion to host extracellular matrix components (Geoghegan and Foster, 2015; Foster, 2019). These include a large family of microbial surface components recognizing adhesive matrix molecules (MSCRAMMs) defined by the presence of two adjacent IgG-like folded domains that participate in ligand binding; proteins containing G5-E repeat domains, such as SasG in *S. aureus* and Aap in *Staphylococcus epidermidis*, which promote cell aggregation; proteins with legume lectin-like domains, such as SraP in *S. aureus*, which binds to N-acetylneuraminic acid-containing glycoproteins in saliva and on the surface of mammalian cells; and many others (Geoghegan and Foster, 2015). The tropisms of these adhesins reflect the host environments to which each species is adapted. For example, *R. gnavus*, which is enriched in some intestinal biofilms, expresses an MSCRAMM, RadA, that binds to intestinal mucins (Maresca et al., 2021), while many oral streptococci and other Gram-positive species produce serine-rich repeat (Srr) proteins and antigen I/II polypeptides that mediate adhesion to saliva-coated surfaces (Nobbs et al., 2011). Surface-associated lipoproteins can also promote adhesion of streptococci to salivary glycoproteins, collagen, or other bacteria (Kolenbrander and London, 1993; Jenkinson, 1994; Bober et al., 2011). Similarly, the intestinal pathogen *C. difficile* expresses a lipoprotein that promotes its adhesion to intestinal epithelial cells (Kovacs-Simon et al., 2014), although this study did not distinguish between binding directly to the epithelial cells versus binding to surface-associated secreted molecules such as mucins.

Gram-negative bacteria also produce a variety of non-pilus surface-associated proteins that mediate adhesion and/or aggregation. For example, although the primary role of flagella is usually considered to be motility, the flagella of *E. coli* and *P. aeruginosa* also exhibit adhesive interactions with mucins (Lillehoj et al., 2002; Erdem et al., 2007; Troge et al., 2012). In the case of *E. coli*, not only whole purified flagella but also individual flagellin subunits bound mucins *in vitro*, suggesting that the entire filament may be capable of mediating such interactions, although the involvement of the flagellar cap protein, FliD, has not been explicitly ruled out (Erdem et al., 2007). In *P. aeruginosa*, flagellin and the flagellar cap protein have each been reported in different studies to mediate adhesion to mucus (Arora et al., 1998; Lillehoj et al., 2002), with the relative importance of the two components possibly depending on the type of mucins tested. Lectins are another important class of Gram-negative bacterial adhesin, as in Gram-positive bacteria. The surface-associated lectin Fap2 expressed by *F. nucleatum* mediates enhanced adhesion to colorectal adenocarcinomas through its specificity for the polysaccharide Gal-GalNAc, which is overexpressed by the cancer cells—potentially contributing to the frequent enrichment of *F. nucleatum* within colorectal-cancer-associated biofilms (Abed et al., 2016). *Bacteroides* species also express lectin-like adhesins that bind to host-derived glycoconjugates and may contribute to epithelial cell adhesion and/or co-aggregation with other bacterial species (Rogemond and Guinet, 1986; London and Allen, 1990).

Outer membrane proteins can also contribute to adhesion and/or aggregation of Gram-negative bacteria. In many enteric bacteria, including urinary tract pathogens, auto-aggregation that contributes to biofilm formation is mediated at least in part by subgroup of autotransporter proteins, which are a class of outer membrane proteins defined by the presence of a signal peptide, a secreted passenger domain, and translocator domain that forms a pore in the outer membrane (Klemm et al., 2006). Similarly, the autotransporter RadD in *F. nucleatum* mediates co-aggregation with other species of oral bacteria (Nobbs et al., 2011). Autotransporters expressed by *Actinomyces*, on the other hand, promote binding to epithelial cells (Nobbs et al., 2011). Other outer membrane proteins involved in adhesion and/or aggregation include the outer membrane protein FomA, which helps *F. nucleatum* to adhere to saliva-coated surfaces (Nobbs et al., 2011); the outer membrane porin F in *P. aeruginosa*, which promotes adhesion to human alveolar epithelial cells (Azghani et al., 2002); and the outer membrane protein HBP35 in *P. gingivalis* promotes co-aggregation with other oral bacteria (Nobbs et al., 2011). In addition, *Bacteroides* species express a TonB-dependent outer membrane protein that binds to fibronectin (Pauer et al., 2009).

Besides proteinaceous adhesins, certain polysaccharides have been implicated in modulating bacterial aggregation and adhesion. For example, cell-bound N-acetylglucosamine (GlcNAc) polysaccharides produced by *E. coli* and *Staphylococcus* species serve as intercellular adhesins that are required for biofilm formation in certain strains (Wang et al., 2004; Itoh et al., 2005; Brady et al., 2008). The genomic locus required for producing such polysaccharides has also been identified in biofilm-forming opportunistic pathogens such as *K. pneumoniae* and *Burkholderia cepacia* (Skurnik et al., 2012), and contributes to the structural integrity of biofilms formed by the oral pathogen *Aggregatibacter actinomycetemcomitans* (Izano et al., 2008b). The exact nature of the molecular interactions underlying the intercellular adhesive function of GlcNAc polysaccharides remains unclear; however, interactions with proteins are likely either not involved or not required, given that cells in *E. coli* biofilms could be dispersed by hydrolysis of the glycosidic linkages of poly-β-1,6-GlcNAc but not by enzymatic protein digestion (Wang et al., 2004; Itoh et al., 2005). Two other well-known extracellular polysaccharides, Pel and Psl, play strain-specific roles in aggregation of *P. aeruginosa* and adhesion to surfaces that include host epithelial cells and mucin-coated substrates (Vasseur et al., 2005; Ma et al., 2006; Byrd et al., 2010). In addition, several *Streptococcus* species synthesize extracellular glucans and fructans from sucrose (Rozen et al., 2001; Banas and Vickerman, 2003). These polysaccharides play essential roles in aggregation of oral bacteria and adherence to tooth surfaces (Rozen et al., 2001; Tamesada et al., 2004; Koo et al., 2010; Matsumoto-Nakano, 2018). Conversely, extracellular polysaccharides can also inhibit biofilm formation when in the form of a capsule, possibly by masking adhesive bacterial cell surface structures (Béchon et al., 2020).

Compared to many of the biofilm-forming species discussed above, considerably less is known about the factors mediating adhesion or aggregation of mycobacterial lung pathogens. Besides the curli-like pilus mentioned in section 3.1.1, one of the few mycobacterial adhesins described to date is a heparin-binding hemagglutinin expressed by *M. tuberculosis* (Menozzi et al., 1996). This protein promotes mycobacterial auto-aggregation and adhesion to epithelial cells (Menozzi et al., 1996), suggesting a possible role in the formation of the sheet-like putative biofilms seen in some tuberculosis lesions (Nyka and O’Neill, 1970). In addition, the antigen 85 complex of *M. tuberculosis* binds fibronectin with increasing strength under mechanical stress, which may be important for mycobacterial adhesion under the shear forces experienced in the lung (Viljoen et al., 2020). Which other surface-associated factors are involved in mycobacterial adhesion and aggregation, if any, remains unclear.

### 3.2 Components of the biofilm extracellular matrix

Following the initial stages of bacterial aggregation or adhesion to a surface, the secretion of extracellular matrix components plays an important role in biofilm maturation and structural integrity. Biofilm extracellular matrix components have been described from all four classes of biological macromolecules, but most often include polysaccharides, proteins, and extracellular DNA (eDNA).

Numerous extracellular polysaccharides contribute to the biofilm matrix in a taxon-specific manner. In *E. coli*, colanic acid is not required for surface attachment but is essential for the development of complex three-dimensional biofilm structures in certain strains (Danese et al., 2000). Colanic acid is also required for biofilm formation by *S. enterica* serovar Typhimurium on human epithelial cells and chicken intestinal tissue, but not on a plastic surface (Ledeboer and Jones, 2005). Besides colanic acid, some strains of *E. coli* and *Salmonella* produce cellulose, which works in concert with curli fibers to increase biofilm cohesion, elasticity, and stability (Serra et al., 2013). Still other biofilm-associated high-molecular-weight extracellular polysaccharides produced by enteric bacteria have yet to be named or genetically characterized (Bales et al., 2013).

Biofilms formed by non-enteric bacteria are likewise often rich in extracellular polysaccharides. In an animal model of middle ear infections, sialylated lipooligosaccharides were an indispensable component of the extracellular matrix secreted by *H. influenzae* (Jurcisek et al., 2005). In oral bacteria, the glucans that promote adhesion to tooth surfaces also play a key role in the development of three-dimensional multicellular structures (Xiao et al., 2012). Similarly, in *P. aeruginosa*, Psl and Pel polysaccharides not only contribute to the early stages of aggregation and adhesion, but also serve as structural scaffolds in mature biofilms (Colvin et al., 2012). For example, positively-charged Pel can crosslink eDNA through ionic interactions (Jennings et al., 2015). The third major exopolysaccharide produced by *P. aeruginosa*, alginate, affects the three-dimensional structure of mature biofilms at least *in vitro*, although it is not required for biofilm formation (Stapper et al., 2004). Overproduction of alginate confers a mucoid phenotype and is common among strains isolated from chronically-infected CF patients, indicating a likely fitness advantage *in vivo* (Lam et al., 1980; Stapper et al., 2004). The role of alginate within the *P. aeruginosa* biofilm matrix may be primarily protective rather than structural, as it can scavenge reactive oxygen species, hinder phagocytosis, and inhibit killing by cationic antimicrobial peptides (Limoli et al., 2015). Finally, cellulose (or a cellulose-like polysaccharide) is present in the extracellular matrix of mycobacterial biofilms, including biofilms formed *in vitro* by nontuberculous mycobacteria and putative *in vivo* biofilms of *M. tuberculosis* detected in infected human lungs (Chakraborty et al., 2021). Treatment of nontuberculous mycobacterial biofilms with either cellulase or proteinase K resulted in dispersal of the bacterial cells (Chakraborty et al., 2021), suggesting that cellulose plays an important structural role, possibly in concert with extracellular proteins, as is also the case for enteric bacteria.

The proteinaceous components of biofilm matrices are as diverse as those involved in aggregation and adhesion. Such proteins can contribute structurally as the biofilm matures either by forming amyloids (i.e. aggregates with fibrillar morphology and β-sheet secondary structure) and/or by interacting with other extracellular polymeric substances. For example, amyloid curli fibers produced by enteric bacteria not only interact with cellulose, as mentioned above, but also with eDNA (Gallo et al., 2015; Nicastro et al., 2022). Although less well-recognized than curli, phenol soluble modulins (Psm) produced by *S. aureus* assemble into amyloid-like fibrils that stabilize the biofilm structure and promote biofilm resistance to enzymatic degradation, surfactant-mediated dispersal, and mechanical stress (Schwartz et al., 2012). *S. mutans* produces functional amyloids formed by antigen I/II polypeptides and other secreted proteins dependent on sortase-mediated assembly, which promote biofilm formation and are present in dental plaque (Oli et al., 2012). *Pseudomonas* species can also produce a functional amyloid protein, encoded by the *fap* operon, that contributes to biofilm mechanical robustness (Zeng et al., 2015) and promotes *in vivo* aggregation of *P. aeruginosa* in a rat model of lung infection (Beg et al., 2022). Another extracellular protein that contributes to the structural stability of the *P. aeruginosa* biofilm matrix is CdrA, which is an adhesin belonging to a two-partner secretion system (Borlee et al., 2010). CdrA binds to the exopolysaccharides Psl and Pel, potentially cross-linking the polysaccharides or tethering them to the bacterial cell surface (Borlee et al., 2010; Reichhardt et al., 2020). Similarly, oral streptococci express a number of proteins that shape the three-dimensional structure of dental plaque biofilms by binding to glucans (Hazlett et al., 1999; Banas and Vickerman, 2003; Matsumoto-Nakano, 2018). The role of Bap, a biofilm-associated protein first discovered in *S. aureus*, is less clear, but it can form amyloids under certain conditions and is thought to contribute to the biofilm matrix (Cucarella et al., 2001; Taglialegna et al., 2016). Although Bap is not common among clinical isolates of *S. aureus*, several other Gram-positive and Gram-negative pathogens encode orthologs, ranging from *Enterococcus* to *Salmonella* (Cucarella et al., 2001; Toledo-Arana et al., 2001; Latasa et al., 2005). Finally, *S. aureus* expresses a series of lipoproteins that modulate biofilm porosity by binding to eDNA (Kavanaugh et al., 2019).

eDNA has been recognized as a potential constituent of the *in vivo* biofilm matrix since at least the 1970s, when analysis of the “slime” produced by several clinical isolates of *P. aeruginosa* revealed the presence of DNA (Murakawa, 1973). For many years, eDNA in biofilms was thought to be mostly a byproduct of lysed cells with unclear functional significance (Sutherland, 2001). However, a subsequent study revealed that DNase could prevent *in vitro* biofilm formation by *P. aeruginosa*, suggesting an important structural or adhesive role (Whitchurch et al., 2002). eDNA has since been shown to be a major component of the extracellular matrix not only for *P. aeruginosa* but also other bacterial species that form biofilms *in vivo*, including *H. influenzae*, *S. aureus*, and *Streptococcus* species (Jurcisek and Bakaletz, 2007; Izano et al., 2008a; Nur et al., 2013; Liao et al., 2014; Sugimoto et al., 2018). Depending on the species, eDNA found in biofilms may be released through autolysis and/or secreted in extracellular vesicles (Kadurugamuwa and Beveridge, 1995; Steinberger and Holden, 2005; Liao et al., 2014). Notably, a recent study demonstrated that across several different biofilm-forming human pathogens, the eDNA in both *in vitro* and *in vivo* biofilms was present not only as B-DNA—the canonical right-handed helical form—but also as DNase-resistant left-handed Z-DNA as a result of interactions with secreted bacterial DNABII proteins (Buzzo et al., 2021). The proportion of Z-DNA increases as biofilms age, explaining the oft-reported observation that DNase cannot always disperse mature biofilms even though it can disrupt biofilm formation (Buzzo et al., 2021). Thus, Z-DNA plays an important role in biofilm structural stability and resistance to host defenses. DNABII orthologs are ubiquitous in eubacterial genomes (Dey et al., 2017), suggesting that this phenomenon might be broadly applicable across *in vivo* biofilms that incorporate eDNA.

Besides polymers, the biofilm extracellular matrix can also include outer membrane vesicles (OMVs). OMVs are heterogeneous, self-enclosed bilayered structures that form by blebbing off from the outer membrane of Gram-negative bacteria. OMVs have been observed in dental plaque biofilms (Holliday et al., 2015), and transmission electron microscopy revealed that OMVs were universally present within the extracellular matrix of biofilms formed by *P. aeruginosa* under multiple growth conditions, as well as in mixed-species biofilms from environmental samples (Schooling and Beveridge, 2006). Thus, OMVs might broadly be part of the biofilm extracellular matrix whenever Gram-negative bacteria are present. OMVs have been shown *in vitro* to mediate coaggregation of oral bacteria (Kamaguchi and Baba, 1995), and may also perform other biofilm-related functions such as binding aminoglycoside antibiotics and thereby protecting cells in the interior (Schooling and Beveridge, 2006). In addition, OMVs are a major source of lipopolysaccharide within the extracellular matrix and can interact with eDNA via salt-bridging and electrostatic interactions, raising the possibility that they impact biofilm structural and mechanical properties (Schooling and Beveridge, 2006; Schooling et al., 2009).

Finally, while biofilm extracellular matrices characterized in axenic cultures consist exclusively of bacterially-produced components, it is possible that bacteria *in vivo* may sometimes incorporate host-derived polymers into their biofilm structure. For example, in the lower respiratory tract or gastrointestinal tract, mucins might represent a potential extracellular matrix substrate. In addition, eDNA is released by host polymorphonuclear leukocytes (PMNs) during infections to create Neutrophil Extracellular Traps (NETs) that can ensnare planktonic bacteria. Bacterial DNABII proteins can convert host-derived eDNA around the periphery of *in vivo* biofilms into Z-DNA (Buzzo et al., 2021), and exogenous DNA can be incorporated into biofilms *in vitro* (Chiang et al., 2013). Nevertheless, surveys of a small number of clinical samples suggest that host-derived eDNA may not be commonly incorporated into the core structure of *in vivo* biofilms (Alhede et al., 2020; Buzzo et al., 2021), and no direct evidence has been published to date of mucin incorporation into biofilms as a structural component. More generally, given that it is challenging to comprehensively characterize the biofilm matrix in tissue samples, it remains unclear to what extent the understanding of biofilm extracellular matrix components gained from *in vitro* studies may translate to the *in vivo* setting.

### 3.3 Environmental regulators of biofilm formation

The genomic capacity to express surface factors with the right tropisms and extracellular matrix components to provide stability are prerequisites for bacteria to successfully form a biofilm. However, whether a biofilm forms at a particular site in the host also depends on the physiochemical characteristics of the microenvironment. Physical forces that affect biofilm formation have been reviewed in greater depth elsewhere (Renner and Weibel, 2011; Arias and Brito, 2021; Wong et al., 2023), but two major factors that are especially relevant to *in vivo* environments are fluid flow and viscoelasticity of the host substrate.

Fluid flow and the attendant shear forces are prominent features of the gastrointestinal tract, urinary tract, respiratory tract, and heart chambers. High rates of fluid flow can promote bacterial clearance (Granick et al., 2007; Dawes, 2008), while low shear forces, such as those believed to be present in the abnormally thickened mucus layer of CF patients, can promote bacterial aggregation (Crabbé et al., 2008). Yet the relationship between fluid shear and bacterial clearance is not linear. Local increases in shear rate near a surface can actually trap motile bacteria and promote attachment (Crabbé et al., 2008; Rusconi et al., 2014). This phenomenon results from the competing effects of cell alignment with the direction of flow and the stochasticity of bacterial swimming orientation (Rusconi et al., 2014). Some bacteria have also evolved mechanisms for sensing fluid shear, and in the case of *P. aeruginosa*, activation of those sensors upregulates the expression of biofilm-related genes (Alsharif et al., 2015; Rodesney et al., 2017; Sanfilippo et al., 2019). Moreover, certain bacterial adhesins exhibit increased adhesive strength in response to tensile forces, as a result of conformational changes (Arias and Brito, 2021). Such adhesins, known as catch-bonds, likely play a role in the ability of *S. aureus* to colonize heart valves during endocarditis, and the ability of uropathogenic *E. coli* to colonize the bladder epithelium (Arias and Brito, 2021). Thus, the relationship between shear rate and biofilm formation can be non-intuitive, particularly within the range of rates typical of *in vivo* environments.

The viscoelastic properties of mucus can also affect the ability of bacteria to establish a biofilm, which is particularly relevant in the respiratory and gastrointestinal tracts. The thickened mucus found in CF lungs behaves more similarly to an elastic solid whereas normal airway mucus is more similar to a viscous liquid (Matsui et al., 2006). Modeling this difference with isotonically concentrated mucus revealed that the thicker mucus promoted the formation of macrocolonies by *P. aeruginosa* (Matsui et al., 2006). Importantly, such macrocolonies were not formed by a mutant defective for quorum sensing, suggesting that they represented true biofilms and could not simply be explained by physical entrapment of the bacteria (Matsui et al., 2006). Viscoelasticity is also highly relevant in the human distal gastrointestinal tract, where the intestinal epithelium is lined with two mucus layers: a relatively loose outer layer facing the lumen, and a much more compact inner layer adjacent to the epithelium (Johansson et al., 2014). Normally, the high viscosity and/or low porosity of the inner layer prevents the incursion of bacteria even in the absence of flow (Johansson et al., 2014). However, in ulcerative colitis, the inner mucus layer often becomes penetrable to bacteria (Johansson et al., 2014; Post et al., 2019). The reasons for this are not fully understood, but presumably precede the establishment of biofilms adjacent to the epithelium.

Besides physical forces, chemical cues within the host environment can also influence the establishment of a biofilm. For example, the dietary polysaccharide maltodextrin increases the adherence of *E. coli* to epithelial cells and promotes biofilm formation in a type 1 pilus-dependent manner (Nickerson and McDonald, 2012). Other organic compounds found in the distal gastrointestinal tract that modulate the production of adhesins or extracellular matrix factors by *E. coli* include N-acetylglucosamine, short-chain fatty acids, and polyamines such as putrescine and spermidine (Rossi et al., 2017). In addition, bile salts or bile acids—detergent-like molecules secreted by the liver to solubilize ingested lipids—induce *in vitro* biofilm formation by a variety of gut-associated bacteria, from commensal *Bacteroides* species (Pumbwe et al., 2007; Béchon et al., 2022) to pathogenic *E. coli*, *Salmonella*, and *Shigella* (Faherty et al., 2012; Nickerson et al., 2017; Köseoğlu et al., 2019). The mechanisms underlying this phenomenon do not appear to be conserved across species, ranging from upregulation of a specific autotransporter (in *Shigella flexneri*) to an unexpected dependency on a constitutively-expressed extracellular DNase (in *B. thetaiotaomicron*) (Faherty et al., 2012; Béchon et al., 2022). Notably, in a clinical study, patients with detectable intestinal biofilms tended to have higher fecal levels of bile acids, and at least one bile acid was significantly enriched in biofilm-positive biopsies compared to biofilm-negative biopsies (other bile acids were not included in the metabolomics panel) (Baumgartner et al., 2021). Thus, bile acids may play a key role in stimulating biofilm formation by enteric pathogens and members of the gut microbiota *in vivo* as well as *in vitro*.

Inorganic compounds in the host environment also modulate biofilm development and persistence. Iron plays a signaling role in biofilm formation for many species, including *P. aeruginosa*, *E. coli*, *S. aureus*, and *S. mutans* (Francesca et al., 2004; Banin et al., 2005; Johnson et al., 2005; Musk et al., 2005; Wu and Outten, 2009; Lin et al., 2012). However, the relationship between iron levels and biofilm phenotypes varies across species and is not necessarily monotonic; for example, in *P. aeruginosa*, iron limitation interferes with biofilm formation, but excess iron can also be inhibitory (Banin et al., 2005; Musk et al., 2005). Oxygen is another widespread regulator of biofilm formation with taxon-specific effects. Uropathogenic *E. coli* upregulate the expression of type 1 pili in response to oxygen (Floyd et al., 2015), while conversely, *S. mutans* grows but is unable to form a biofilm in the presence of oxygen (Ahn and Burne, 2007). Hypoxia enhances biofilm formation by *S. aureus* (Mashruwala et al., 2017), and *P. aeruginosa* similarly forms larger biofilms when respiring nitrate in the absence of oxygen, compared to aerobic growth with or without nitrate (Yoon et al., 2002)—observations that are highly relevant to the hypoxic, nitrate-replete microenvironment of CF sputum (Grasemann et al., 1998; Worlitzsch et al., 2002). Finally, nitrate itself influences the production of curli and cellulose by uropathogenic *E. coli* (Martín-Rodríguez et al., 2020) and serves as a cue for the dispersal of *Salmonella* biofilms (Miller et al., 2022b). Both *E. coli* and *Salmonella* encounter nitrate during the course of infection, as human urine contains nitrate (Radomski et al., 1978), and nitrate levels rise in the intestine during inflammation (Rivera-Chávez and Bäumler, 2014).

## 4 Impacts of tissue-associated biofilms on human health

Biofilms can have numerous impacts on human health depending on their location and composition. Here we review several effects that are supported by experimental evidence or clinical observations. Not all of these effects are mechanistically well-understood, and more may remain to be discovered.

### 4.1 Decreased efficacy of antibiotic treatment

One widely-accepted consequence of biofilm formation during infections is decreased efficacy of antibiotic treatments, which can delay the clearance of pathogens and lead to chronic infections. In fact, although not an apples-to-apples comparison, the antibiotic concentration required to eradicate a biofilm can be more than 1000-fold higher than the minimum inhibitory concentration for the same strain in planktonic form (Ceri et al., 1999). Even very small, actively growing biofilm-like clusters of bacteria containing as few as 150 cells can exhibit reduced antibiotic susceptibility compared to cells growing at lower densities (Connell et al., 2010).

Multiple mechanisms are thought to underlie the decreased antibiotic susceptibility of biofilm-dwelling bacteria. First, electrostatic or other interactions with components of the extracellular matrix can limit the diffusion of certain antibiotics within a biofilm and consequently their ability to access bacteria in the interior. For example, negatively-charged alginate and eDNA can bind positively-charged antibiotics such as aminoglycosides (Hatch and Schiller, 1998; Chiang et al., 2013). Second, the three-dimensional architecture of a biofilm generates gradients of nutrients, pH, terminal electron acceptors, and waste products (Stewart and Franklin, 2008). Consequently, different subpopulations within a biofilm may experience starvation, oxygen limitation, or other physiological stresses, leading to reduced metabolic activity and slower growth. This inherently limits the efficacy of many antibiotics that target the synthesis of cellular components or depend on the membrane potential for uptake into the cell (Tack and Sabath, 1985; Stewart, 2002). Third, such stresses can activate adaptive response pathways that prime bacteria to tolerate subsequent stresses induced by antibiotics (Mulcahy et al., 2008; Nguyen et al., 2011; Bernier et al., 2013; Wilton et al., 2016). Fourth, spontaneous mutation rates may be increased within biofilms as a result of oxidative stress or other factors, leading to *de novo* acquisition of antibiotic resistance (Driffield et al., 2008; Saint-Ruf et al., 2014).

Importantly, not all of the mechanisms discussed above are necessarily in play for every biofilm. In the study that reported the surprising presence of morphologically similar biofilms in both chronic and acute lower respiratory infections, biofilm-encased bacteria in the acute infections appeared to be more metabolically active than the bacteria in chronic infections, based on the per-cell ribosome content (Kolpen et al., 2022). Although the antibiotic susceptibility of those bacteria was not directly tested, this observation suggests that microenvironmental differences between chronic and acute infections, and not just the presence of a biofilm *per se*, might play a critical role in modulating antibiotic susceptibility *in vivo*.

### 4.2 Evasion of the host immune response

In addition to enhancing bacterial survival during antibiotic treatment, biofilms can also offer protection against the host immune response to infection. The sheer size and dense matrix of a biofilm can present a physical obstacle to engulfment by immune cells, as biofilms that are otherwise impervious to phagocytosis by macrophages become sensitized upon mechanical disruption (Thurlow et al., 2011). Other biofilm immune evasion mechanisms are taxon-specific. For example, *P. aeruginosa* biofilms respond to the presence of PMNs by secreting rhamnolipids, which stick to the extracellular matrix and induce necrosis in any immune cells that make contact (Alhede et al., 2009). In addition, alginate protects *P. aeruginosa* biofilms from phagocytosis by macrophages (Leid et al., 2005). Biofilm formation also has a protective effect for *Staphylococcus* species, but the extent of protection varies depending on the circumstances. One study indicated that fluid shear increases leukocyte penetration of *S. aureus* biofilms, compared to biofilms grown under static conditions, but the invading leukocytes were nevertheless unable to phagocytose bacteria within the biofilm (Leid et al., 2002). On the other hand, a different study demonstrated that PMNs can penetrate and clear *S. aureus* biofilms by phagocytosis, although aged biofilms were less susceptible than biofilms that were only a few days old (Günther et al., 2009). *S. aureus* biofilms can also limit invasion and phagocytosis by macrophages (Thurlow et al., 2011), possibly by inducing macrophage cytotoxicity (Scherr et al., 2015), while the large quantity of poly-N-acetylglucosamine within the *Staphylococcus* biofilm extracellular matrix serves as a decoy that prevents antibody-mediated killing (Cerca et al., 2006). Extracellular polysaccharides similarly protect *S. mutans* against killing by neutrophils, an effect that was enhanced for surface-attached bacteria compared to those in suspension (Steinberg et al., 1999).

Interestingly, *S. aureus* biofilms *in vivo* decreased the levels of cytokines responsible for immune cell recruitment and activation (Thurlow et al., 2011), and the polysaccharides produced by *S. mutans* attenuated neutrophil release of reactive oxygen species (Steinberg et al., 1999). Furthermore, in a rabbit ear wound model, infections with predominantly biofilm-associated *S. aureus* provoked a diminished inflammatory response compared to infections that also contained abundant planktonic bacteria, despite similar levels of viable bacteria in both types of infections (Gurjala et al., 2011). Thus, besides posing a physical obstacle or secreting cytotoxic factors, some bacterial biofilms also dampen the overall magnitude of the immune response via pathways that have not been fully elucidated.

### 4.3 Delayed wound healing

In soft tissue wounds, biofilms delay the healing process. Evidence for this comes not only from the correlation between the chronicity of a wound and the presence of biofilms (James et al., 2008)—which does not indicate the direction of causality—but also from experiments with several animal models of wound healing. For example, in a diabetic mouse model, infection of wounds with pre-formed biofilms of *P. aeruginosa* delayed complete healing by two to four weeks compared to uninfected controls (Zhao et al., 2012). Similarly, in a non-diabetic mouse model, inoculation of punch wounds with pre-formed *Staphylococcus* biofilms resulted in slower re-epithelialization (Schierle et al., 2009). Other studies in which biofilms were allowed to develop over time within wounds have further supported the role of biofilms in delayed healing by clarifying the impact of biofilm presence versus simply bacterial presence. In one study, infection of rabbit ear wounds with a biofilm-impaired Pel- and Psl-deficient mutant of *P. aeruginosa* resulted in improved healing and lower expression of inflammatory cytokines, compared to infection with the wildtype parent strain, even though both strains colonized the wounds to similar levels (Seth et al., 2012). In another study, infection of porcine burn wounds with hyperbiofilm-forming and biofilm-deficient mutants of *S. aureus* revealed that the hyperbiofilm-forming strain attenuated re-epithelialization compared to the wildtype parent strain (Roy et al., 2020). Moreover, both the hyperbiofilm-former and biofilm-competent wildtype strain induced a significant loss of collagen in granulation tissue compared to the biofilm-deficient mutant (Roy et al., 2020). Multispecies biofilms may have even more severe impacts on wound healing than monospecies biofilms, as inoculation of porcine wounds with a mixed population of biofilm-forming *P. aeruginosa* and *S. aureus* uniquely suppressed the expression of a keratinocyte growth factor and further delayed re-epithelialization, compared to inoculation with only one strain or the other (Pastar et al., 2013). Finally, clinical observations suggest that anti-biofilm wound care strategies, such as regular debridement to physically disrupt biofilms, can improve patient outcomes for chronic wounds, further supporting the causal role of biofilms in delayed healing (Wolcott and Rhoads, 2008; Metcalf and Bowler, 2013).

### 4.4 Dental disease

Biofilms in the form of dental plaque are not a marker for disease, but when left unchecked, plaque can undergo dysbiotic shifts that lead to the deterioration of oral health (Frédéric et al., 2018). For example, biofilm-dwelling *S. mutans* is thought to be one of the major culprits driving the formation of dental caries (Marsh, 2010). Caries form when tooth enamel is eroded by the acid produced by bacterial fermentation of carbohydrates, particularly sucrose (Leme et al., 2006). Sucrose is also a substrate for extracellular matrix production by *S. mutans* (Banas and Vickerman, 2003), and this three-dimensional matrix in turn facilitates the development of acidic pockets within the plaque biofilm (Xiao et al., 2012).

Like caries, periodontal disease is inextricably linked to plaque biofilms. Early colonizers provide a physical foothold for later colonizers, and the community shifts that occur as plaque matures are associated with the development of gingival inflammation (Kolenbrander et al., 2010; Frédéric et al., 2018). Moreover, the pathogenic potential of oral bacteria is influenced by contact-dependent interspecies communication and signaling by short-range diffusible metabolites, both of which are enhanced within biofilms (Kolenbrander et al., 2010). Mechanical disruption of plaque biofilms is the current standard of care for periodontal disease, and a recent clinical study confirmed that this intervention significantly decreased the abundance of disease-associated species, while taxa that are typically associated with oral health either increased in abundance or remained unchanged (Johnston et al., 2021). However, while it is important for restoring oral health in periodontal patients, mechanical disruption of plaque can also transiently introduce bacteria into the bloodstream and thereby enable biofilms to disseminate to other parts of the body, as in some cases of infective endocarditis (Larsen and Fiehn, 2017).

### 4.5 Promotion of cancer development

A relatively recent development in the field of biofilm research is the finding that biofilms in the gastrointestinal tract may promote carcinogenesis. Multiple studies have reported a correlation between colorectal cancer and the incidence of bacterial biofilms on the intestinal epithelium (Dejea et al., 2014; Drewes et al., 2017), and biofilm-positive normal intestinal tissues display markers of a pro-oncogenic state, including increased epithelial cell proliferation and reduced expression or altered localization of E-cadherin (Dejea et al., 2014). In addition, some strains of *E. coli* and *Bacteroides fragilis* secrete oncogenic toxins (Wu et al., 2009; Pleguezuelos-Manzano et al., 2020), and such bacteria were enriched in biofilms adhering to pre-cancerous lesions in patients with familial adenomatous polyposis (Dejea et al., 2018). However, perhaps the most direct evidence that biofilms can be pro-oncogenic comes from a study in which microbial consortia from biofilm-positive or biofilm-negative human colonic biopsies were inoculated into three different murine models of carcinogenesis (Tomkovich et al., 2019). In that study, regardless of whether the biofilm-positive biopsies were from colon cancer patients or healthy controls, all biofilm consortia significantly increased the number of colonic tumors that formed over a 12-week period in both germ-free and specific-pathogen-free mice, compared to inocula prepared from biofilm-negative biopsies. Moreover, in contrast to the biofilm-negative biopsy communities, all biofilm consortia at least partially invaded the mucus layer of the distal colon within 12 weeks when inoculated into the germ-free mice, suggesting that certain bacteria are inherently more capable of breaching the mucus barrier (Tomkovich et al., 2019). The biofilm consortia also significantly induced mucosal infiltration by Th17 and other IL-17+ cells, as well as by myeloid cells, in agreement with prior studies indicating a role for IL-17 in tumorigenesis (Wu et al., 2009; Housseau et al., 2016; Tomkovich et al., 2019).

Interestingly, a case-control study in humans revealed a correlation between both the presence and severity of periodontal disease and the incidence of oral cancer (Komlós et al., 2021), suggesting that pathogenic oral biofilms might similarly induce pro-oncogenic changes in surrounding tissues. Consistent with this hypothesis, incubation of oral squamous cell carcinoma cell lines with periodontal pathogens increased cell migration and tumorsphere formation, whereas commensal oral bacteria had no effect (Kamarajan et al., 2020). In addition, *H. pylori*, which forms biofilms in the gastric mucosa, is a well-known risk factor for gastric cancer. Although a causal link between *H. pylori* biofilm formation and the initiation or progression of gastric cancer has yet to be definitively demonstrated, strains of *H. pylori* containing a specific cytotoxin-associated gene pathogenicity island are not only more proficient at forming biofilms *in vitro* (Wong et al., 2016), but are also associated with increased risk of gastric cancer (Blaser et al., 1995; Huang et al., 2003). Thus, bacterial biofilms on mucosal tissues beyond the distal gastrointestinal tract might more broadly represent a cancer risk factor.

### 4.6 Instigation or exacerbation of autoimmune disease

Another potential effect of some biofilms is the development of autoimmune disorders. Such disorders arise in a small percentage of patients following infections with enteric pathogens such as *Salmonella*, *Shigella*, and *E. coli*, all of which can produce amyloid-forming curli as part of their biofilm extracellular matrix (Miller et al., 2022a). Curli fibers bind tightly to eDNA in biofilms, and the resulting curli-DNA complexes potently stimulated the immune system and induced the production of autoantibodies both in a mouse model of lupus and in wildtype mice (Gallo et al., 2015). Similarly, infection of mice with curli-producing invasive *Salmonella* triggered the production of autoantibodies and induced joint inflammation (Miller et al., 2020). Systemic exposure of immune cells to the curli-DNA complexes appears to be necessary for the stimulation of autoimmunity, as infection with non-invasive curli-producing *Salmonella* or oral administration of curli-DNA complexes did not have the same effect (Miller et al., 2020). Notably, a recent clinical study reported that flares in lupus patients correlated with increased serum levels of anti-curli/DNA antibodies, and that these episodes also correlated with the presence of bacteria in the patients’ urine, suggesting that similar mechanisms may be at work in humans (Pachucki et al., 2020). Moreover, autoimmune sequelae are also seen in a minority of patients infected with *P. aeruginosa*, *M. tuberculosis*, or *S. aureus* (Miller et al., 2022a). Like enteric bacteria, all of these species produce amyloidogenic proteins that could potentially form complexes with eDNA (see section 3.2).

### 4.7 Colonization resistance

Although tissue-associated biofilms in humans have primarily been associated with disease states, not all biofilms may have a negative impact on the host. In the oral context, it has been proposed that in healthy dental plaque, commensal bacteria serve as a barrier to colonization by oral pathogens (Marsh, 2010). In support of this hypothesis, certain oral commensal species have been shown to antagonize the growth and/or adhesion of taxa associated with periodontitis, or to interfere with biofilm-formation by *S. mutans* (Hoogmoed et al., 2008; Tamura et al., 2009; Begić et al., 2023). Colonization resistance has also been studied in the context of the distal gastrointestinal tract. Biofilms formed by commensal enteric bacteria *in vitro* resist incursion by enteric pathogens (Re et al., 2013), and pre-colonization of mice with specific combinations of commensal *E. coli* strains prevented subsequent colonization by a pathogenic strain of *E. coli* (Leatham et al., 2009; Maltby et al., 2013). Pre-colonization with a common probiotic strain was also able to prevent epithelial damage by pathogenic *E. coli* in human intestinal organoids (Pradhan and Weiss, 2020). Whether biofilm formation is necessary for *in vivo* intestinal colonization resistance remains unclear. However, the loss of key adhesins involved in biofilm formation interfered with the ability of probiotic *E. coli* to stably colonize the mouse gut (Lasaro et al., 2009), suggesting that adherence to the mucosal surface might be a prerequisite for sustained beneficial effects, including colonization resistance.

## 5 Discussion

### 5.1 Major open questions and limitations of current knowledge

The study of biofilms in human tissues has come a long way since the time of van Leeuwenhoek. Nevertheless, given that much of the existing mechanistic insight into tissue-associated biofilms has been acquired in the context of single-species studies with canonical pathogens, numerous gaps remain in our understanding of where, when, why, and how biofilms form in the human body. Biofilms *in vivo* are often polymicrobial, yet how interspecies interactions contribute to the course of biofilm development is not well-understood in most cases. In addition, relatively little is known about the regulation and mechanisms of biofilm formation by commensal bacteria, as well as how such biofilms impact health and disease.

Taking the distal gastrointestinal tract as an example, there is no unifying explanation for the observation that biofilms on the intestinal epithelium are more prevalent in various disease states, yet are not always absent in healthy controls. Are the biofilms that are occasionally found in healthy controls precursors to disease, or is there such a thing as a commensal—or even beneficial—biofilm in the gut? And to what extent do biofilms present in disease states actively drive pathology, as opposed to representing a symptom of an underlying disturbance? Similar questions also apply to other organ systems. For example, given that some clinically non-infected patients have putative biofilms in their upper respiratory tract, and that some asymptomatic women have vaginal biofilms composed of species typically associated with disease, are there characteristics that better enable certain people to keep potentially pathogenic biofilms in check? Such characteristics might include variants in genes related to the immune system, or the presence of beneficial bacteria that antagonize pathogenic species. It is also possible that there is a tipping point, perhaps in terms of tissue coverage or length of association, beyond which biofilms are more likely to cause symptoms.

The true prevalence of biofilms in healthy states is also unclear, as biofilm distribution can be patchy and it is impractical to microscopically survey entire organs. While the difference in frequency of detection implies that biofilms are indeed more prevalent in many disease states across different tissue types, it is possible that relatively small, localized biofilms may be more common than is currently appreciated. Because conclusive evidence for biofilms in regions such as the distal gastrointestinal tract, upper respiratory tract, and urogenital tract is based on small biopsies, it is difficult to distinguish between a difference in actual prevalence (i.e. the percentage of people with any biofilm) and a difference in tissue coverage (i.e. the percentage of the tissue surface that is associated with a biofilm). Furthermore, the method of tissue processing can greatly affect the likelihood of detecting a biofilm. For example, when biopsies of the intestinal epithelium are fixed in formalin, adherent biofilms are completely lost; instead, fixation in nonaqueous Carnoy’s solution or Methacarn, which preserve the mucus layer and the biofilm extracellular matrix, appears to be a prerequisite for biofilm detection in this context (Swidsinski et al., 2005b; Dassanayake et al., 2020). It is possible that the prevalence of biofilms on other mucosal tissues may have been underestimated by studies that used standard aqueous fixatives.

Another barrier to understanding the prevalence of tissue-associated biofilms is that some tissues that might harbor biofilms, such as the small intestine, are difficult to access in living patients. Others, such as the urinary tract, are not commonly subjected to biopsies, especially in healthy individuals. With regard to the latter, recent sequencing-based studies on samples obtained by transurethral catheterization or suprapubic aspiration have revealed the presence of a distinct bladder microbiome in healthy people, from which live bacteria can be grown using non-standard culture conditions (Thomas-White et al., 2016). Moreover, experiments performed nearly forty years ago revealed that bacteria obtained from the vagina or distal urethra of healthy women could adhere to human uroepithelial cells *in vitro*, and some species were able to competitively exclude uropathogenic bacteria (Chan et al., 1984; Chan et al., 1985). Thus, it is not implausible that biofilms might exist in the bladder or urethra beyond the context of urinary tract infections, but the requirement for invasive sampling will hinder the testing of this hypothesis.

Much also remains to be understood about the process of and triggers for biofilm formation within the host environment. Is the formation of biofilms *in vivo* driven primarily by intrinsic characteristics of their constituent taxa, or are permissive host characteristics and/or environmental triggers also necessary? Most likely, all three factors contribute to some degree, but the extent of their contribution may vary across different body sites. In the lower respiratory tract, the species that are commonly detected in biofilms are also prolific biofilm-formers *in vitro*, indicating the importance of intrinsic bacterial features. Yet it is clear that host factors and environmental changes, such as the thickening of mucus in CF patients, affect the likelihood of biofilm establishment and persistence, even though not all factors are well-understood. Particularly in the case of factors such as fluid shear that have a non-intuitive, non-linear relationship to biofilm formation, further studies will be required in order to better understand how disease-related changes affect biofilm development by different species and in different contexts.

Intrinsic bacterial characteristics likely also contribute to biofilm formation in the distal gastrointestinal tract, given that biofilm communities transplanted from human biopsies can invade the distal colonic mucus layer in mice (Tomkovich et al., 2019). Importantly, however, that experiment was performed in germ-free mice, which at baseline have a more penetrable inner mucus layer than conventional mice (Johansson et al., 2015). In this respect, germ-free mice bear more resemblance to ulcerative colitis patients than to healthy humans (Johansson et al., 2014), raising the possibility that defects in the mucus structure are also a predisposing factor for biofilm formation. The repeated observation that epithelial biofilms are more commonly detected in the proximal colon compared to the distal colon further suggests that environmental triggers are likely to be important, yet the influence of extrinsic factors on regulation of biofilm formation remains almost entirely unknown for many of the taxa that have been detected in intestinal biofilms. Even in the case of known environmental triggers, such as bile acids, the underlying signal transduction pathways have yet to be elucidated. In addition, basic information is lacking about the range of fluid shear rates within the human intestinal environment and the influence of solid-induced shear forces generated by fecal contents (Hinman et al., 2021).

Finally, with the exception of oral biofilms, little information is available about the dynamics of biofilm formation and persistence *in vivo*, due to the invasive nature of sample collection for most tissues. The ability to conduct time-course studies *in situ* in the oral context has revealed reproducible trends in community composition over time. Does ecological succession similarly occur in other tissue-associated multispecies biofilms, such as those found in the intestine or chronic wounds, and if so, are there generalizable principles? Relatedly, how persistent are biofilms, both in health and in disease, and when, why, and how do they disperse? Drivers and mechanisms of biofilm dispersal have been studied *in vitro* and reviewed elsewhere (Rumbaugh and Sauer, 2020), but direct observations *in vivo* are challenging for both technical and practical reasons.

### 5.2 Importance of high-fidelity experimental models

Given the difficulty of accessing most tissues for *in situ* biofilm studies, addressing the questions outlined above will require the development of advanced experimental systems that model the human tissue environment as accurately as possible. The most common methods for studying biofilms, such as microtiter plate assays or flow chambers (Azeredo et al., 2017), fail to capture important aspects of the host environment, which can severely affect the human relevance of study outcomes (Bjarnsholt et al., 2013). For example, lactoferrin, a host-derived innate defense molecule, is effective at preventing *P. aeruginosa* biofilm formation on abiotic surfaces in well-oxygenated flow chambers (Singh et al., 2002), but culturing *P. aeruginosa* in concentrated mucus from human airways revealed a complete lack of efficacy of lactoferrin in preventing the formation of suspended aggregate-type biofilms resembling those found in CF patients (Matsui et al., 2006). Furthermore, while animal models can enable biofilms to be studied in a more holistic *in vivo* context, bacterial tropisms can be host-specific, and common model organisms such as mice exhibit significant differences with humans in terms of anatomy, diet, bile acid composition, native microbiota, respiratory secretions, and so on (Benahmed et al., 2014; Nguyen et al., 2015).

Although no model system is perfect, two organ systems for which relatively advanced *in vitro* models are already available are the respiratory tract and the gastrointestinal tract. With regard to the respiratory tract, different aspects of the CF environment have been modeled individually, such by using artificial sputum media to mimic the nutritional composition of CF sputum (Fung et al., 2010), or culturing bacteria in agar suspensions to study the effects of aggregation and metabolic gradients on antibiotic susceptibility (Spero and Newman, 2018). More recently, an *ex vivo* pig lung model for infections of CF bronchioles showed promise in overcoming many of the limitations of simpler *in vitro* models and mouse models (Harrington et al., 2020). Progress has also been made towards modeling respiratory infections of non-CF patients, such as through the use of differentiated primary human airway cells cultured at an air-liquid interface (Hasan et al., 2018). As for the gastrointestinal tract, complex *in vitro* models have been developed that simulate many aspects of the oral environment, including saliva, fluid flow, and surfaces that resemble natural tooth enamel, although challenges remain in modeling bacterial interactions with gingival tissue (Luo et al., 2022). Primary-tissue-derived models of the human gastric mucosa and intestinal epithelium have also taken shape over the last decade, including three-dimensional organoids that largely recapitulate the cell-type diversity of the native tissue; apical-out organoids in which the orientation of the polarized epithelium is inverted for easier experimental access; two-dimensional organoid-derived monolayers; and organoid-derived microfluidic systems (Sato et al., 2011; Fujii et al., 2018; Co et al., 2019; Kim et al., 2019; Sontheimer-Phelps et al., 2020). Such models are increasingly used for the study of host-microbe interactions, and some have demonstrated the capacity to support co-cultures for several days (Jalili-Firoozinezhad et al., 2019; Shin et al., 2019). Microfluidic systems are particularly attractive because they enable the incorporation of shear forces. However, further iterations may be required to establish the utility of organoid-derived models for studying biofilm interactions with the host epithelium (Puschhof et al., 2021).

### 5.3 Concluding remarks

It is an exciting time to be studying the impact of biofilms on human health and disease. A significant body of evidence has accumulated regarding the existence of biofilms in diverse tissues. For many of the most common biofilm-forming species, reductionist studies have led to a detailed understanding of the molecular factors involved in biofilm development and persistence. Moreover, intriguing correlations between biofilm presence and multiple disease states hint that developing biofilm-targeting drugs could impact the treatment and prevention of not only chronic infections, as is now well-accepted, but also other intractable conditions ranging from inflammatory bowel disease to cancer. While many questions remain to be explored, the advent of complex *in vitro* experimental models will provide researchers with new tools to untangle how the host environment affects biofilm phenotypes, and how specific biofilm components trigger host responses. This in turn will improve our ability to detect and manipulate tissue-associated biofilms *in situ*, with potentially wide-ranging implications for human health.

## Author contributions

EP wrote the article with input from M-WT. All authors contributed to the article and approved the submitted version.
